# Use of benzo analogs to enhance antimycotic activity of kresoxim methyl for control of aflatoxigenic fungal pathogens

**DOI:** 10.3389/fmicb.2014.00087

**Published:** 2014-03-07

**Authors:** Jong H. Kim, Noreen Mahoney, Kathleen L. Chan, Bruce C. Campbell, Ronald P. Haff, Larry H. Stanker

**Affiliations:** Foodborne Toxin Detection and Prevention Research Unit, Western Regional Research Center, USDA-ARSAlbany, CA, USA

**Keywords:** aflatoxin, antioxidant system, *Aspergillus*, cell wall integrity, chemosensitization, octylgallate, strobilurin, veratraldehyde

## Abstract

The aim of this study was to examine two benzo analogs, octylgallate (OG) and veratraldehyde (VT), as antifungal agents against strains of *Aspergillus parasiticus* and *A.flavus* (toxigenic or atoxigenic). Both toxigenic and atoxigenic strains used were capable of producing kojic acid, another cellular secondary product. *A. fumigatus* was used as a genetic model for this study. When applied independently, OG exhibits considerably higher antifungal activity compared to VT. The minimum inhibitory concentrations (MICs) of OG were 0.3–0.5 mM, while that of VT were 3.0–5.0 mM in agar plate-bioassays. OG or VT in concert with the fungicide kresoxim methyl (Kre-Me; strobilurin) greatly enhanced sensitivity of *Aspergillus* strains to Kre-Me. The combination with OG also overcame the tolerance of *A. fumigatus* mitogen-activated protein kinase (MAPK) mutants to Kre-Me. The degree of compound interaction resulting from chemosensitization of the fungi by OG was determined using checkerboard bioassays, where synergistic activity greatly lowered MICs or minimum fungicidal concentrations. However, the control chemosensitizer benzohydroxamic acid, an alternative oxidase inhibitor conventionally applied in concert with strobilurin, did not achieve synergism. The level of antifungal or chemosensitizing activity was also “compound—strain” specific, indicating differential susceptibility of tested strains to OG or VT, and/or heat stress. Besides targeting the antioxidant system, OG also negatively affected the cell wall-integrity pathway, as determined by the inhibition of *Saccharomyces cerevisiae* cell wall-integrity MAPK pathway mutants. We concluded that certain benzo analogs effectively inhibit fungal growth. They possess chemosensitizing capability to increase efficacy of Kre-Me and thus, could reduce effective dosages of strobilurins and alleviate negative side effects associated with current antifungal practices. OG also exhibits moderate antiaflatoxigenic activity.

## Introduction

Controlling fungi that produce hepato-carcinogenic aflatoxins in crops, such as tree nuts, corn, peanuts, etc., is problematic as effective commercial fungicides for treating aflatoxigenic fungi are very limited (Roze et al., 2013). Aflatoxins are secondary metabolites produced mainly by the filamentous fungi *Aspergillus flavus* and *A. parasiticus*. Significant amounts of harvested crop can be made unsuitable for sale and consumption as a result of aflatoxin contamination. Very low level (parts per billion) of aflatoxin contamination can have a perniciously negative effect on food safety and economic value of a number of crops from year to year (Campbell et al., 2003). Therefore, effective methods are continually needed for control of aflatoxigenic fungal pathogens.

Strobilurins are widely used agricultural fungicides. Strobilurins were initially identified in the fungus *Strobilurus tenacellus*, and were synthetically developed into several subgroups, such as kresoxim methyl (Kre-Me), azoxystrobin, pyraclostrobin, etc. (Bartlett et al., 2002 and references therein). The molecular target for strobilurins is the *bc*_1_ complex (complex III; ubiquinol-cytochrome *c* oxidoreductase, EC 1.10.2.2) in the mitochondrial respiratory chain (MRC). Strobilurins specifically bind to the Q_P_ (Q_O_) center of cytochrome *b* (Bartlett et al., 2002 and references therein), resulting in the inhibition of MRC.

Development of fungal resistance to conventional antifungal agents is a global agricultural issue (Ghannoum and Rice, 1999; Wood and Hollomon, 2003; Possiede et al., 2009; Cools and Hammond-Kosack, 2013). For example, agricultural fields receiving continuous applications of the fungicide, strobilurin, resulted in the development of insensitivity of fungi to this fungicide (Keinath, 2009). Noteworthy is that if strobilurin-containing fungicides are applied at suboptimal time-points of fungal growth, these fungicides can actually potentiate mycotoxin production in fungi (Ellner, 2005).

Similar type of fungicide-potentiation of mycotoxin production was documented in *Penicillium verrucosum*, a filamentous fungal pathogen producing the mycotoxin, citrinin (Schmidt-Heydt et al., 2013). When *P. verrucosum* was treated with the fungicide “Rovral” (Iprodione), an inhibitor of DNA/RNA biosynthesis and cell division, the growth of fungi was decreased. However, a concomitant strong induction of citrinin biosynthesis also occurred in fungi with the same treatment (Schmidt-Heydt et al., 2013). Altogether, studies indicated that certain conventional fungicides could stimulate secondary metabolism, especially mycotoxin production, in fungal pathogens. Therefore, effective strategies are urgently needed to overcome counterproductive repercussions of fungicides currently in use.

Recent studies have shown that safe, natural phenolic compounds or their structural derivatives (e.g., benzo derivatives) could act as potent antifungal or antimycotoxigenic agents (Beekrum et al., 2003). For example, vanillic or caffeic acid not only inhibited the growth of *Fusarium verticillioides*, but also reduced its production of the mycotoxin, fumonisin (Beekrum et al., 2003). Natural benzo derivatives could also effectively inhibit the growth of *A. fumigatus*, *A. flavus*, *A. terreus*, and *Penicillium expansum* (Kim et al., 2011). These fungi are causative agents of human invasive aspergillosis or are producers of mycotoxins, including aflatoxin, patulin, gliotoxin, etc. The redox-active natural phenolic agents can be potent redox cyclers that inhibit fungal growth by disrupting cellular redox homeostasis (and thus triggering cellular oxidative stress) (Guillen and Evans, 1994; Jacob, 2006). For defense, the fungal antioxidant system, such as oxidative signaling pathway, plays an important role for fungal tolerance to those phenolic agents (Kim et al., 2011).

Genes involved in stress-signaling pathways are also important for fungal virulence, pathogenesis and protection from oxidative burst exerted by the host (Washburn et al., 1987; Hamilton and Holdom, 1999; Clemons et al., 2002; de Dios et al., 2010). Oxidative stress signals sensed by a fungal cell are incorporated into the upstream mitogen-activated protein kinase (MAPK) pathway, which regulates the expression of the downstream response genes (such as antioxidant enzyme genes) detoxifying the stress (Miskei et al., 2009). In *A. fumigatus*, SakA and MpkC are orthologous MAPKs to *Saccharomyces cerevisiae* Hog1p, which plays a key role in countering oxidative stress (Toone and Jones, 1998; Lee et al., 2002; Xue et al., 2004; Reyes et al., 2006; Miskei et al., 2009).

Chemosensitization is a strategy where combined application of certain types of compounds along with a conventional fungicide/drug enhances the effectiveness of the conventional agents (Niimi et al., 2004; Agarwal et al., 2012; Campbell et al., 2012). Noteworthy is that certain benzo derivatives possessed antifungal chemosensitizing capability. In our prior study, co-application of antifungal agents with chemosensitizing benzo derivatives, such as 2-hydroxy-5-methoxybenzaldehyde, greatly enhanced the efficacy of antifungal agents (Kim et al., 2011). Thus, chemosensitization could lead to lowering dosages of conventional fungicides/drugs required for control of pathogens, especially drug-resistant strains. Collectively, these studies showed the potential for safe, natural phenolics to serve as effective antifungal and/or antimycotoxigenic agents.

In nature, in addition to oxidative stress, high temperature (heat) stress is another type of environmental challenge that many microbes face, which also triggers “signaling cascades” in fungal cells (Morano et al., 2012). Heat treatment is also a strategy to prevent contamination by food spoilage fungi in foods (Dagnas and Membré, 2013). In corn, artificial drying of maize kernels with high temperatures is one of the postharvest practices to prevent fungal growth and aflatoxin production (Hawkins et al., 2005). A prior study showed that heat treatment at 70°C significantly reduced the maize kernel infection of *A. flavus*. However, heat treatment had no effect on aflatoxin concentration, which reflects the heat stability of the mycotoxin (Hawkins et al., 2005). Also, heat treatment can result in deterioration of the quality of the crop (seed breakage, viability, etc.). Therefore, development of new alternative strategies, which warrant early intervention of mycotoxin production/fungal growth as well as the quality of harvested crops, is necessary.

In this study, we investigated the role of two benzo derivatives, octylgallate (OG) and veratraldehyde (VT), currently used as food additives, as antifungal agents against strains of *A. flavus, A. parasiticus*, or *A. fumigatus*. OG and VT are generally regarded as safe (GRAS) reagents (FDA, 2011). We also evaluated antifungal chemosensitizing capacities of OG and VT, especially for overcoming strobilurin resistance of *Aspergillus* MAPK gene deletion mutants (*sakA*Δ, *mpkC*Δ). Kre-Me, containing (*E*)-methyl methoxyiminoacetate group in the structure, was tested as an exemplary strobilurin. Kre-Me is currently applied in the agricultural field for control of various fungal diseases caused by ascomycete, basidiomycete, oomycete, etc., while it exhibited high toxicity to the agriculturally important insects, bees (Bartlett et al., 2002). Our results showed that OG could serve as a potent antifungal chemosensitizer to Kre-Me for controlling *Aspergillus* strains.

## Materials and methods

### Microbial strains and culture conditions

Microbial strains used in this study are summarized in Table 1. *Aspergillus* strains were cultured on potato dextrose agar (PDA) at 30°C, except otherwise noted in the text. Yeast strains, wild type (WT) and gene deletion mutants of *Saccharomyces cerevisiae* (Table 1), were cultured on Synthetic Glucose (SG; Yeast nitrogen base without amino acids 0.67%, glucose 2% with appropriate supplements: uracil 0.02 mg mL^−1^, amino acids 0.03 mg mL^−1^) or Yeast Peptone Dextrose (YPD; Bacto yeast extract 1%, Bacto peptone 2%, glucose 2%) medium at 30°C.

**Table 1:** **Microbial strains used in this study**.

	**Characteristics**	**Source/references**
***Aspergillus***		
*A. flavus* 3357	Plant pathogen (aflatoxin), Human pathogen (aspergillosis), Reference toxigenic (aflatoxin-producing) strain used for genome sequencing	NRRL^a^, *Aspergillus* Comparative Database
*A. flavus* 4212	Plant pathogen (aflatoxin), Human pathogen (aspergillosis)	NRRL
*A. flavus* 21882	Atoxigenic (aflatoxin non-producing) strain, A pesticide active ingredient displacing toxigenic fungus	NRRL, *Aspergillus flavus* NRRL 21882 Fact Sheet
*A. flavus* 18543	Atoxigenic strain, A pesticide active ingredient displacing toxigenic fungus	NRRL, Ehrlich and Cotty (2004)
*A. parasiticus* 5862	Plant pathogen (aflatoxin)	NRRL
*A. parasiticus* 2999	Plant pathogen (aflatoxin)	NRRL
*A. fumigatus* AF293	Human pathogen (aspergillosis), Parental strain, Reference clinical strain used for genome sequencing	Xue et al. (2004); *Aspergillus* Comparative Database
*A. fumigatus sakA*Δ	Human pathogen (aspergillosis), Mitogen-Activated Protein Kinase (MAPK) gene deletion mutant derived from AF293	Xue et al. (2004)
*A. fumigatus mpkC*Δ	Human pathogen (aspergillosis), MAPK gene deletion mutant derived from AF293	Reyes et al. (2006)
***Saccharomyces***		
*S. cerevisiae* BY4741	Model yeast, Parental strain (Mat a *his3*Δ*1 leu2*Δ*0 met15*Δ*0 ura3*Δ*0*)	(SGD)^b^
*S. cerevisiae bck1*Δ	MAPK kinase kinase mutant derived from BY4741	SGD
*S. cerevisiae slt2*Δ	MAPK mutant derived from BY4741	SGD

a *NRRL, National Center for Agricultural Utilization and Research, USDA-ARS, Peoria, IL, USA*.

b *Genome Database. Available online: http://www.yeastgenome.org (accessed on 10 January 2014)*.

### Chemicals

**Figure 1:**
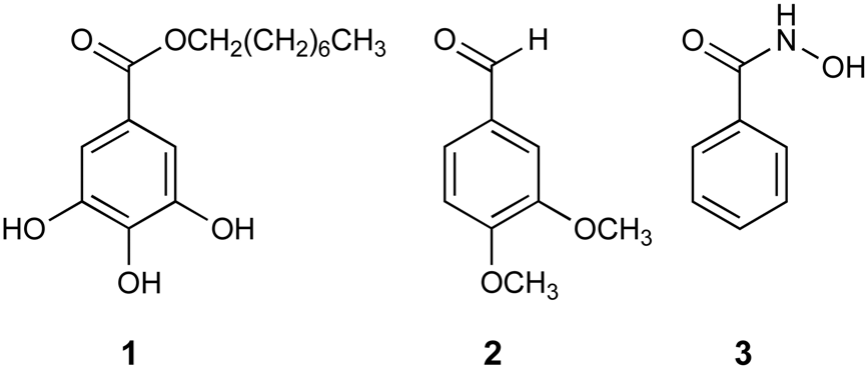
**Structures of benzo analogs used in this study**. (1) octylgallate (OG); (2) veratraldehyde (VT); (3) benzohydroxamic acid (BHAM).

Antifungal compounds, kresoxim methyl (Kre-Me; strobilurin), octylgallate (octyl 3,4,5-trihydroxybenzoic acid; OG), veratraldehyde (3,4-dimethoxybenzaldehyde; VT), benzohydroxamic acid (BHAM) (Figure 1), were procured from Sigma Co. (St. Louis, MO, USA). Each compound was dissolved in dimethylsulfoxide (DMSO; absolute DMSO amount: <2% in media) before incorporation into culture media (except for those plates used in aflatoxin assays; see below). Throughout this study, control plates (No treatment) contained DMSO at levels equivalent to that of cohorts receiving antifungal agents, within the same set of experiments.

### Susceptibility testing

#### Agar plate-bioassay in aspergillus

In agar plate-bioassays, susceptibility of filamentous fungi to OG, VT, or Kre-Me, alone or in combination, were measured based on percent radial growth of treated compared to control fungal colonies (see Figures and Tables for test concentrations). The percent inhibition of growth was calculated using the Vincent equation (Vincent, 1947) [% inhibition = 100 (*C*−*T*)/*C*; where *C* = diameter of fungal colony on control plate (receiving only DMSO), and *T* = diameter of fungal colony on the treated plate]. Minimum inhibitory concentration (MIC) values on agar plates were based on triplicate assays and defined as the lowest concentration of agent where no fungal growth was visible on the plate. For the above assays, fungal conidia (5 × 10^3^) were diluted in phosphate buffered saline and applied as a drop onto the center of PDA plates with or without antifungal compounds. Growth was observed for 5–7 days.

#### CLSI liquid bioassay in aspergillus

To determine the precise level of chemosensitizing activities of OG or BHAM (0.00625, 0.0125, 0.025, 0.05, 0.1, 0.2, 0.4, 0.8 mM) to Kre-Me (0.5, 1, 2, 4, 8, 16, 32 μg mL^−1^) in the strains of *Aspergillus*, checkerboard bioassays (triplicate) (0.4 × 10^4^–5 × 10^4^ CFU mL^−1^) were performed in microtiter wells using a broth microdilution method (in RPMI 1640 medium; Sigma Co., St. Louis, MO, USA), according to protocols outlined by the Clinical and Laboratory Standards Institute (CLSI) M38-A (CLSI, 2008). OG was chosen since this compound showed much higher antifungal activity than VT (see Results). For comparison, BHAM, an alternative oxidase (AOX) inhibitor conventionally used in combination with strobilurin, was also included as a reference chemosensitizer in this test. RPMI 1640 medium was supplemented with 0.03% L-glutamine and buffered with 0.165 mM 3-[*N-*morpholino] propanesulfonic acid. MICs, lowest concentration of agents showing no visible fungal growth in microtiter wells (200 μL per well), were assessed after 48 and 72 h. Minimum fungicidal concentrations (MFCs), lowest concentration of agents showing ≥99.9% fungal death, were determined following completion of MIC assays by spreading entire volumes of microtiter wells (200 μ L) onto individual PDA plates, and culturing for another 48 and 72 h. Compound interactions, Fractional Inhibitory Concentration Indices (FICIs) and Fractional Fungicidal Concentration Indices (FFCI), were calculated as follows: FICI or FFCI = (MIC or MFC of compound A in combination with compound B/MIC or MFC of compound A, alone) + (MIC or MFC of compound B in combination with compound A/MIC or MFC of compound B, alone). Interactions were defined as: “synergistic” (FICI or FFCI ≤ 0.5) or “indifferent” (FICI or FFCI > 0.5–4) (Odds, 2003).

### Growth recovery test in *Aspergillus* strains treated with high temperatures: agar plate-bioassay

Agar plate-based bioassay was performed to evaluate differential susceptibility of *Aspergillus* strains to high temperatures. First, fungal conidia (5 × 10^3^) were spotted on PDA (see above for method), and were initially incubated at four different temperatures (moderate: 30, 35°C; high: 45, 55°C). Triplicate PDA plates were then removed from each temperature (30, 35, 45, or 55°C) at day 1, 2, 3, and 4, and were transferred to 30°C for additional 6, 5, 4, and 3 days of growth, respectively, resulting in a total of 7 days of incubation for each treatment (i.e., 1 day growth at 55°C + 6 days growth at 30°C = Total 7 days growth, 2 days growth at 55°C + 5 days growth at 30°C = Total 7 days growth, 3 days growth at 55°C + 4 days growth at 30°C = Total 7 days growth, 4 days growth at 55°C + 3 days growth at 30°C = Total 7 days growth). For controls, *Aspergillus* strains were grown solely at respective temperature (30, 35, 45, or 55°C) for 7 days. The level of growth recovery at 30°C was evaluated based on fungal radial growth as described above.

### Growth recovery test in cell wall integrity mutants of *Saccharomyces cerevisiae* treated with OG: yeast dilution bioassay

To determine the effect of OG on the cell wall-integrity system of fungi, sorbitol recovery tests were performed using Petri plate-based yeast dilution bioassays. Ten-fold diluted, i.e., 10^0^ (1 × 10^6^ cells), 10^−1^, 10^−2^, 10^−3^, 10^−4^, 10^−5^, strains of *S. cerevisiae* BY4741 (WT), *bck1*Δ and *slt2*Δ were spotted on: (1) SG only, (2) SG + caffeine (5 mM; a positive control for cell wall disruption) or SG + OG (0.03, 0.04, 0.05, 0.06 mM) (Testing sensitivity of *bck1*Δ and *slt2*Δ mutants to compounds), and (3) SG + sorbitol (0.5 M) + caffeine or OG (Testing growth recovery of *bck1*Δ and *slt2*Δ mutants by sorbitol). Cell growth was monitored for 5–7 days. If the growth score on the sorbitol-containing medium was higher than that on the “no sorbitol” medium, OG was considered to negatively affect cell wall integrity.

### Aflatoxin assays

For aflatoxin analysis, spore suspensions (200 spores) were inoculated onto the center of PDA plates. Cells were grown at 30°C for 7 days with or without OG (OG concentration: 0.025, 0.05, 0.1 mM; added directly into PDA), with each treatment in triplicate. Aflatoxins were quantitated as described previously (Rodriguez and Mahoney, 1994). Aflatoxin standard solutions (AFB_1_, AFB_2_, AFG_1_, AFG_2_), used for the quantification of aflatoxins from OG-treated samples, were prepared as described in AOAC 971.22 (AOAC International, 2005).

### Kojic acid assays

To examine whether the cellular secondary-product metabolism other than aflatoxin production is normal in the atoxigenic strains used in this study (namely, *A. flavus* 21882, 18543), we determined kojic acid (KA) production in these atoxigenic strains. *A. flavus* 3357 and 4212, toxigenic strains, were used as controls for KA production. Fungal spores (1 × 10^5^ spores per 100 μL, 0.05% Tween 80) were inoculated into 50 mL potato dextrose broth, and were incubated at 30°C without shaking. KA production was measured from the culture media using an HPLC system consisting of a degasser, autosampler, quaternary pump, and photodiode array detector (Agilent 1100, Santa Clara, CA USA). Culture media aliquots of 1 mL were filtered through 0.45 μm sterile nylon 25 mm syringe filters (Fisherbrand, Thermo Fisher Scientific, Waltham, MA, USA), with injection volumes of 20 μL on a 4.6 × 250 mm Inertsil 5 μm ODS-3 column (GL Sciences, Torrance, CA, USA) using an isocratic mobile phase consisting of MeOH/0.1% H_3_PO_4_ (25:75, v/v) at a flow rate of 1 mL per minute. KA quantitation was based on UV detection at 265 nm. HPLC quantification of KA was linear in the range of 0.02–4.00 μg per 20 μL, with a retention time of 5.4 min.

### Statistical analysis

Statistical analysis (student's *t*-test) was performed based on “Statistics to use” (Kirkman, 2014), where *p* < 0.05 was considered significant.

## Results

### Susceptibility of *Aspergillus* strains to OG or VT: agar plate-bioassay

As shown in Table 2, OG possessed much higher antifungal activity compared to VT. The average MICs of OG was 0.4 mM, while that of VT was higher than 4.8 mM (viz. more than 10 times higher antifungal activity of OG).

**Table 2:** **Differential sensitivity of *Aspergillus* strains to OG, VT, or high temperature (55°C)**.

**Strains**	**OG (mM)**	**VT (mM)**	**High temperature recovery from 55°C (Days)^a^**
*A. flavus* 3357	0.40	>5.0^b^	0
*A. flavus* 4212	0.40	>5.0	0
*A. flavus* 21882	0.50	>5.0	0
*A. flavus* 18543	0.45	>5.0	1
*A. parasiticus* 5862	0.40	>5.0	1
*A. parasiticus* 2999	0.50	>5.0	1
*A. fumigatus* AF293	0.35	4.5	4
*A. fumigatus sakA*Δ	0.30	4.5	4
*A. fumigatus mpkC*Δ	0.30	4.5	4
Average	0.40	>4.8	1.7
*t*-test	*p* < 0.005^c^	–	–

*Concentrations (mM) are MICs examined on PDA plates*.

a *Maximum day(s) of incubation at 55°C, which can result in growth recovery of fungi at 30°C*.

b *VT was tested up to 5.0 mM. For statistical calculation (student's t-test) purpose, 10.0 mM (doubling of 5.0 mM) was used*.

c *Student's t-test for paired data (MIC_OG_) was vs. MIC_VT_ determined in nine strains*.

Results also indicated differential susceptibility (namely, different MIC level) of *Aspergillus* strains to the treatments, where: (1) All *A. fumigatus* strains (WT, *sakA*Δ, *mpkC*Δ) were more sensitive to either OG or VT compared to other *Aspergillus* strains (*p* < 0.05 for OG, *p* < 0.005 for VT), 2) *A. fumigatus* MAPK mutants (*sakA*Δ, *mpkC*Δ) were more sensitive to OG compared to the WT (*p* < 0.005). Although MICs of VT for all *A. fumigatus* strains were similar (MIC_VT_: 4.5 mM), the level of growth of MAPK mutants was also lower than the WT (see below; Table 3). Thus, results indicated MAPK mutants lack the defense mechanism to protect fungal cells from the toxicity generated by redox-active benzo derivatives, (3) *A. flavus* 21882 and *A. parasiticus* 2999 were more tolerant to OG compared to other *Aspergillus* strains (*p* < 0.05), (4) *A. flavus* 3357, 4212 and *A. parasiticus* 5862 showed similar levels of susceptibility to OG, and (5) *A. flavus* 3357, 4212, 21882, 18543 and *A. parasiticus* 5862, 2999 also showed similar levels of susceptibility to VT (up to 5 mM).

**Table 3:** **Responses of *A. fumigatus* WT and MAPK mutants to the treatments of benzo derivatives w/o or w/Kre-Me (25 μM)^a^**.

**VT (mM)**	**AF293 %**	***sakA*Δ %**	***mpkC*Δ %**	**OG (mM)**	**AF293 %**	***sakA*Δ %**	***mpkC*Δ %**
**w/o Kre-Me**							
0.0	100	100	100	0.000	100	100	100
0.5	100	100	96	0.025	86	70	74
1.0	100	98	96	0.050	70	60	66
1.5	100	96	94	0.100	54	48	52
2.0	100	92	92	0.150	46	38	42
2.5	100	92	92	0.200	36	24	28
3.0	96	84	86	0.250	28	few	few
3.5	75	69	72	0.300	few	**0**	**0**
4.0	few^b^	few	few	0.350	**0**	**0**	**0**
4.5	**0**	**0**	**0**	0.400	**0**	**0**	**0**
5.0	**0**	**0**	**0**	0.450	**0**	**0**	**0**
**w/Kre-Me**							
0.0	100	104	109	0.000	100	104	109
0.5	83	96	96	0.025	52	few	few
1.0	70	78	83	0.050	**0**	**0**	**0**
1.5	61	74	74	0.100	**0**	**0**	**0**
2.0	52	65	70	0.150	**0**	**0**	**0**
2.5	43	57	65	0.200	**0**	**0**	**0**
3.0	**0**	48	48	0.250	**0**	**0**	**0**
3.5	**0**	few	few	0.300	**0**	**0**	**0**
4.0	**0**	**0**	**0**	0.350	**0**	**0**	**0**
4.5	**0**	**0**	**0**	0.400	**0**	**0**	**0**
5.0	**0**	**0**	**0**	0.450	**0**	**0**	**0**

a *Data shown in the table are % radial growth of fungi compared to control (no treatment). SD < 5%. No visible cell growth (0%) is in bold*.

b *Few: Only few colonies appeared*.

### Susceptibility of *Aspergillus* to high temperatures

**Figure 2:**
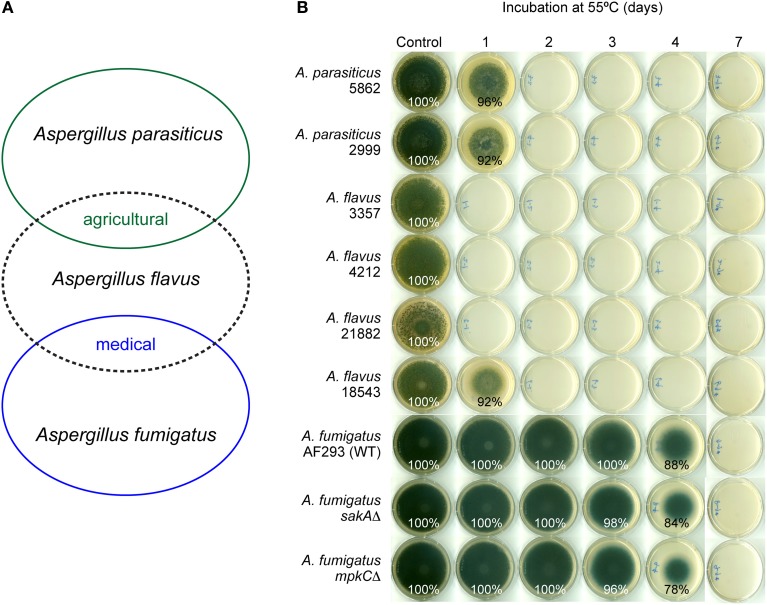
**Differential sensitivity of *Aspergillus* strains to high temperature (55°C). (A)** Scheme showing *A. flavus* shares agro-infectivity with *A. parasiticus*, while it shares human-infectivity with *A. fumigatus*. **(B)** Fungal plate bioassay showing *A. fumigatus* strains were more tolerant to high temperature (55°C) compared to *A. flavus* or *A. parasiticus*. Control, Incubation at 30°C for 7 days; 1–7, Incubation at 55°C for 1, 2, 3, 4, or 7 days, then growth recovery at 30°C for 6, 5, 4, 3, or 0 days, respectively. Fungal growth is indicated as percent relative to control. Unlabeled plates showed no growth (0%).

Heat responses of *Aspergillus* strains were compared to their responses to benzo derivatives. *Aspergillus* strains were treated with moderate (30, 35°C) to high (45, 55°C) temperatures, and were then transferred to 30°C for growth recovery. From the pathogenicity perspective, *A. flavus* shares agro-infectivity with *A. parasiticus*, while *A. flavus* shares human-infectivity with *A. fumigatus* (Figure 2A). As shown in Figure 2B, *A. fumigatus* strains, both WT and MAPK mutants, were much more tolerant to high temperature (55°C), compared to other *Aspergillus* strains examined. For example, *A. fumigatus* strains did not germinate on PDA when they were maintained at 55°C for 7 days. However, *A. fumigatus* treated with heat (55°C) for 1–4 days could recover the growth after incubation at 30°C, while *A. flavus* 3357, 4212 and 21882 could not recover their growth even after 1 day-heat treatment at 55°C (see Table 2 for summary). The remaining *Aspergillus* strains (18543, 5862, 2999) showed growth recovery only with 1 day-heat treatment at 55°C. *A. fumigatus* MAPK mutants were marginally more sensitive to heat (55°C) (i.e., 2–10% less radial growth during recovery w/ 3–4 day-treatment at 55°C) compared to the WT (Figure 2B).

The order of susceptibility of *Aspergillus* strains to high temperature (55°C) (higher susceptibility to lower susceptibility) was: *A. flavus* 3357, 4212, 21882 > *A. flavus* 18543, *A. parasiticus* 5862, 2999 > *A. fumigatus* strains. Therefore, like in benzo treatments, *Aspergillus* strains showed differential susceptibility to high temperature (55°C), where *A. fumigatus* strains were the most heat-tolerant species among *Aspergillus* strains tested. However, there was no direct correlation in fungal responses between benzo and heat treatments, indicating the mechanism of antifungal action of benzo analog(s) is considered to be different from that of heat treatment (see Table 2). [*Aspergillus* strains were also sensitive to 45°C. However, all *Aspergillus* strains recovered their growth after transfer from 45 to 30°C (see Figure S1 for exemplary bioassay with *A. flavus*). As expected, *Aspergillus* strains grew normally at the moderate temperatures (30 and 35°C) (Figure S1)].

### Susceptibility of *A. fumigatus* to chemosensitization

Chemosensitizing capability of OG or VT to Kre-Me was tested in *A. fumigatus* strains by using agar plate-bioassays. Results showed that: (1) When Kre-Me was co-applied with VT, the MIC of VT was lowered from 4.5 mM to 3.0 mM_MAPK_ or 4.0 mM_WT_, respectively (Table 3), and (2) When Kre-Me was co-applied with OG, the MIC of OG was lowered from 0.3 mM_MAPK_ or 0.35 mM_WT_ to 0.05 mM_MAPK and WT_. Therefore, both OG and VT possessed chemosensitizing capability to Kre-Me in *A. fumigatus* strains, where OG possessed much higher chemosensitizing capacity (viz. needed ~10 times lower concentration for chemosensitization; *p* < 0.005) than VT (Table 3).

Noteworthy is that the *A. fumigatus* MAPK mutants were more tolerant to Kre-Me (Table 3), where: (1) The level of radial growth of MAPK mutants with independent treatment of Kre-Me was marginally greater (4–9% more growth) than the WT, and (2) When VT was co-applied with Kre-Me (25 μM), MIC_VT_ was 3.0 mM for the WT, while that of MAPK mutants was 4.0 mM (namely, higher concentration of VT is needed compared to the WT) during this chemosensitization (Table 3). Therefore, although VT-mediated chemosensitization was achieved in all *A. fumigatus* strains, results showed relatively higher tolerance of MAPK mutants to “Kre-Me + VT” compared to the WT [see below (Table 4) for relative tolerance of MAPK mutants to “Kre-Me + BHAM” compared to the WT]. However, Kre-Me tolerance of *A. fumigatus* MAPK mutants was completely abolished by OG (Table 3).

**Table 4:** **Antifungal chemosensitization of OG (mM) or BHAM (mM) to Kre-Me (μg mL^−1^), tested against *Aspergillus* strains: summary of CLSI-based microdilution bioassays (48 h)^a^**.

	**Compounds**	**MIC alone**	**MIC combined**	**FICI**	**Compounds**	**MFC alone**	**MFC combined**	**FFCI**
***Aspergillus* STRAINS OG**								
*A. flavus* 3357	Kre-Me	>32^b^	2	**0.2**	Kre-Me	>32	0.5	**0.3**
	OG	0.2	0.025		OG	0.8	0.2	
*A. flavus* 4212	Kre-Me	>32	2	**0.2**	Kre-Me	>32	0.5	**0.3**
	OG	0.2	0.025		OG	0.8	0.2	
*A. flavus* 21882	Kre-Me	>32	2	**0.2**	Kre-Me	>32	0.5	**0.1**
	OG	0.2	0.025		OG	>0.8	0.2	
*A. flavus* 18543	Kre-Me	>32	2	**0.2**	Kre-Me	>32	2	**0.1**
	OG	0.2	0.025		OG	>0.8	0.1	
*A. parasiticus* 5862	Kre-Me	>32	4	**0.2**	Kre-Me	>32	4	**0.2**
	OG	0.2	0.025		OG	>0.8	0.2	
*A. parasiticus* 2999	Kre-Me	>32	4	**0.2**	Kre-Me	>32	1	**0.1**
	OG	0.2	0.025		OG	>0.8	0.2	
*A. fumigatus* AF293	Kre-Me	>32	1	**0.1**	Kre-Me	>32	0.5	**0.5**
	OG	0.1	0.0125		OG	0.2	0.1	
*A. fumigatus sakA*Δ	Kre-Me	>32	1	**0.1**	Kre-Me	>32	0.5	**0.5**
	OG	0.1	0.0125		OG	0.1	0.05	
*A. fumigatus mpkC*Δ	Kre-Me	>32	1	**0.1**	Kre-Me	>32	0.5	**0.5**
	OG	0.1	0.0125		OG	0.1	0.05	
Mean	Kre-Me	64.00	2.11	**0.1**	Kre-Me	64.00	1.11	**0.2**
	OG	0.17	0.02		OG	0.93	0.14	
*t*-test^c^	Kre-Me	–	*p* < 0.005	–	Kre-Me	–	*p* < 0.005	–
	OG	–	*p* < 0.005	–	OG	–	*p* < 0.005	–
***Aspergillus* STRAINS BHAM**								
*A. fumigatus* AF293	Kre-Me	>32	4	0.6	Kre-Me	>32	>32	2.0
	BHAM	>0.8^d^	0.8		BHAM	>0.8	>0.8	
*A. fumigatus sakA*Δ	Kre-Me	>32	16	0.8	Kre-Me	>32	>32	2.0
	BHAM	>0.8	0.8		BHAM	>0.8	>0.8	
*A. fumigatus mpkC*Δ	Kre-Me	>32	16	0.8	Kre-Me	>32	>32	2.0
	BHAM	>0.8	0.8		BHAM	>0.8	>0.8	
All other strains	Kre-Me	>32	>32	2.0	Kre-Me	>32	>32	2.0
	BHAM	>0.8	>0.8		BHAM	>0.8	>0.8	
Mean	Kre-Me	64.00	46.67	1.6	Kre-Me	64.00	64.00	2.0
	BHAM	1.60	1.33		BHAM	1.60	1.60	
*t*-test	Kre-Me	–	*p* < 0.1	–	Kre-Me	–	ND^e^	–
	BHAM	–	*p* < 0.1	–	BHAM	–	ND	–

a *MIC, Minimum inhibitory concentration; MFC, Minimum fungicidal concentration; FICI, Fractional Inhibitory Concentration Indices; FFCI, Fractional Fungicidal Concentration Indices. Synergistic FICIs and FFCIs are in bold*.

b *Kre-Me was tested up to 32 μg mL^−1^. For calculation purpose, 64 μg mL^−1^ (doubling of 32 μg mL^−1^) was used*.

c *Student's t-test for paired data (combined, i.e., chemosensitization) was vs. mean MIC or MFC of each compound (alone, i.e., no chemosensitization) determined in strains*.

d *BHAM was tested up to 0.8 mM. For calculation purpose, 1.6 mM (doubling of 0.8 mM) was used*.

e *ND, Not determined (neutral interaction)*.

**Figure 3:**
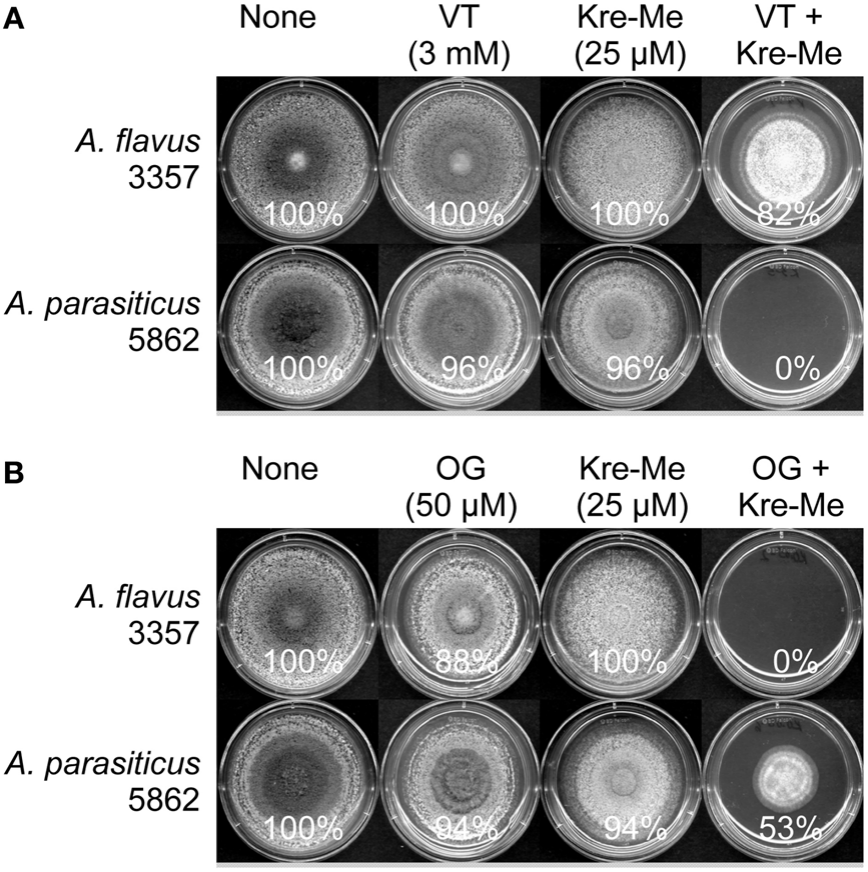
**Differential sensitivity of *A. flavus* 3357 and *A. parasiticus* 5862 to co-treatment of Kre-Me with VT or OG. (A)** *A. parasiticus* 5862 was more sensitive to “Kre-Me + VT” compared to *A. flavus* 3357. **(B)** *A. flavus* 3357 showed higher sensitivity to “Kre-Me + OG” compared to *A. parasiticus* 5862. Results show differential sensitivity of each strain to different combinations of compounds.

Co-application of OG or VT with Kre-Me also enhanced growth inhibition in toxigenic strains of *Aspergillus* (*A. flavus* 3357, *A. parasiticus* 5862) (viz. either complete growth inhibition or reduced radial growth, depending on types of strains/combination of compounds; Figure 3). For example, the growth of *A. parasiticus* 5862 was completely inhibited by Kre-Me + VT, while a similar level of growth inhibition in *A. flavus* 3357 could be achieved by Kre-Me + OG (Figure 3). Independent treatment of each compound alone at the same concentration did not achieve such a level of growth inhibition. Thus, results indicated differential susceptibility of each strain to different combinations of antifungal agents, where 3357 showed higher susceptibility (complete growth inhibition) to Kre-Me + OG, whereas 5862 was more susceptible to Kre-Me + VT (complete growth inhibition). As observed in *A. fumigatus*, OG possessed much higher antifungal potency in toxigenic *Aspergillus* strains compared to VT (namely, 50 μM_OG_ vs. 3 mM_VT_, respectively, for achieving chemosensitization).

**Figure 4:**
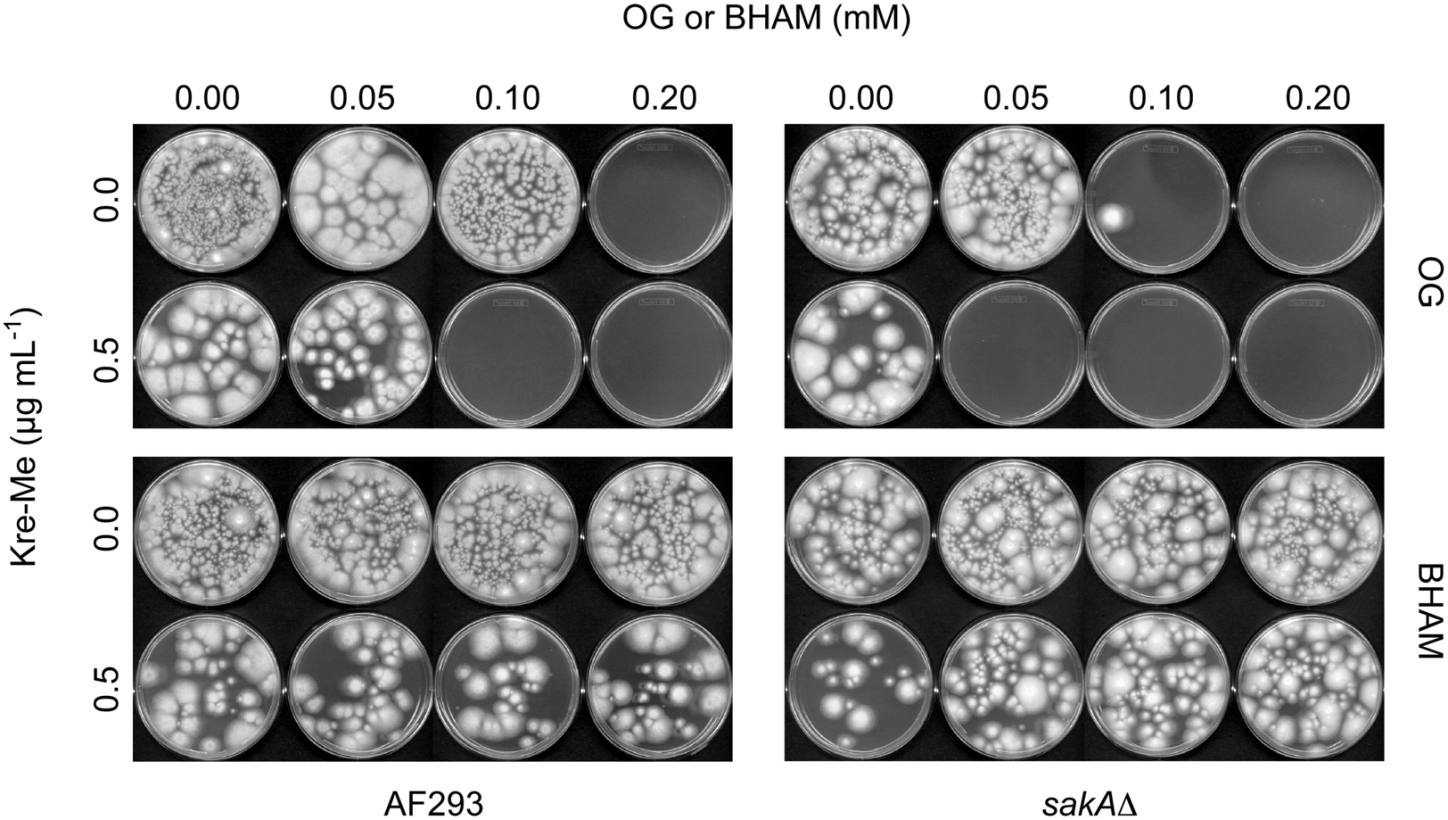
**Chemosensitization test in *A. fumigatus*: Kre-Me + OG or BHAM**. Results shown here are the determination of MFCs of antifungal agents. Co-application of Kre-Me (0.5 μg mL^−1^) with OG (0.10 or 0.05 mM for *A. fumigatus* WT or *sakA*Δ, respectively), completely inhibited the growth of *A. fumigatus*, while individual treatment of each compound, alone, at the same concentrations allowed the growth of fungi. The *sakA*Δ mutant was also more sensitive (viz. required lower concentration of OG) to the chemosensitization than the WT (AF293), indicating that the antioxidant system of *Aspergillus* plays an important role for fungal tolerance to the chemosensitization. On the other hand, co-application of Kre-Me with BHAM (conventional antifungal chemosensitizer disrupting AOX) at the same concentrations resulted in survival of *A. fumigatus* strains. Similar result was observed in *A. fumigatus mpkC*Δmutant (Figure data not shown).

**Figure 5:**
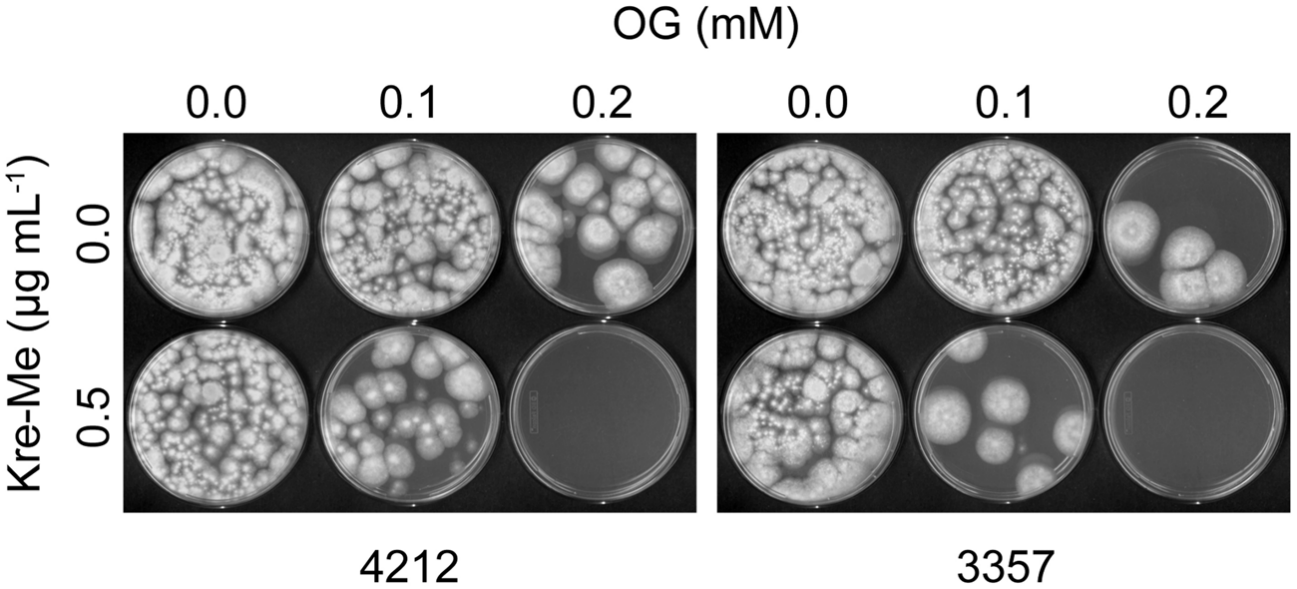
**Chemosensitization test in aflatoxigenic *Aspergillus*: Kre-Me + OG**. Exemplary MFC bioassay showing co-application of Kre-Me (0.5 μg mL^−1^) with OG (0.2 mM) completely inhibited the growth of aflatoxigenic *A. flavus* 3357 and 4212.

In CLSI liquid bioassay, co-application of Kre-Me with OG completely inhibited the growth of *Aspergillus* strains (100% killing in MFC testing), where independent treatment of each compound alone at the same concentrations allowed the survival of fungi (Figures 4, 5). For MICs, “synergistic” FICIs (FICI ≤ 0.5; see Materials and Methods for calculations) were found between OG and Kre-Me for all *Aspergillus* strains (Table 4). For MFCs, “synergistic” FFCIs (FFCI ≤ 0.5) were also achieved by co-application of OG with Kre-Me for all *Aspergillus* strains tested (Table 4). Although there was increased antifungal activity of BHAM and Kre-Me when co-applied in *A. fumigatus* strains (WT, *sakA*Δ, *mpkC*Δ) (FICI = 0.6–0.8; Table 4), no calculated synergism was found in any of the *Aspergillus* strains tested with BHAM + Kre-Me (Table 4). As with VT + Kre-Me (see above), *A. fumigatus* MAPK mutant also showed higher tolerance to “BHAM + Kre-Me” chemosensitization, where the value of MIC_COMBINED_ of Kre-Me was 4 μg mL^−1^ for WT, while that for MAPK mutants was 16 μg mL^−1^, respectively, (viz. four times higher tolerance to Kre-Me) (Table 4). Noteworthy is that certain fungi with mutation(s) in the MAPK signaling pathway can also escape toxicity of the commercial fungicide fludioxonil (Kojima et al., 2004).

### Growth recovery test in cell wall integrity mutants of *S. cerevisiae* treated with OG

**Figure 6:**
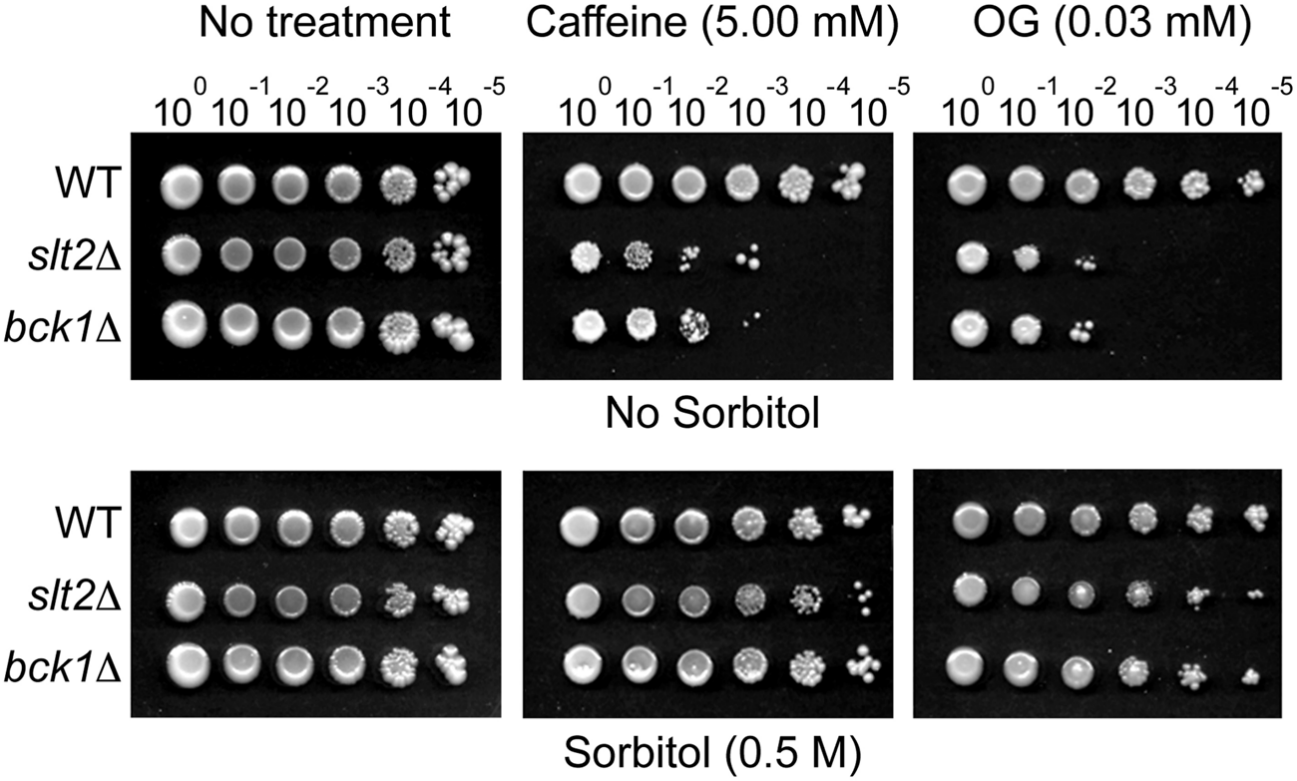
**Yeast cell-dilution bioassay showing sensitivity of *S. cerevisiae slt2*Δ and *bck1*Δ mutants to caffeine (5 mM; control) and OG (0.03 mM) was remediated by sorbitol**. Similar remediation by sorbitol was found from treatment with higher concentration (0.04, 0.05, 0.06 mM) of OG (results not shown).

In sorbitol remediation bioassay, sensitivity of *slt2*Δ and *bck1*Δ to OG was alleviated by sorbitol. The growth rate of *slt2*Δ and *bck1*Δ on sorbitol-containing media was 100–1000 times higher for caffeine or OG, respectively, compared to controls without sorbitol (Figure 6). Thus, the remediation by sorbitol indicates disruption of cell wall/membrane integrity in fungi is one contributing mechanism of how OG generates chemosensitization.

### Effect of OG on aflatoxin production

When toxigenic strains of *A. flavus* and *A. parasiticus* were treated with OG (tested at 25, 50, 100 μM), the highest reduction in aflatoxin production was observed at 25 or 50 μM of OG, depending on types of strains. Aflatoxin production in OG-treated plates was reduced 4–30% compared to non-treated control plates (Table 5).

**Table 5:** **Antiaflatoxigenic activity of OG in *A. flavus* 3357, 4212, *A. parasiticus* 2999 (OG at 25 μM) and *A. parasiticus* 5862 (OG at 50 μM)**.

		***A. flavus* 3357**	***A. flavus* 4212**	***A. parasiticus* 2999**	***A. parasiticus* 5862**
AFB_1_	No OG	5.920 ± 0.816	6.170 ± 0.816	16.100 ± 1.325	16.100 ± 2.904
	OG	5.260 ± 0.663	5.460 ± 0.561	14.900 ± 1.119	15.200 ± 1.271
	% Reduction	−11%	−12%	−7%	−6%
AFB_2_	No OG	0.090 ± 0.020	0.031 ± 0.010	0.290 ± 0.046	0.290 ± 0.112
	OG	0.066 ± 0.015	0.026 ± 0.005	0.260 ± 0.036	0.240 ± 0.033
	% Reduction	−27	−16	−10	−17
AFG_1_	No OG	ND^a^	ND	4.030 ± 0.612	4.030 ± 1.581
	OG	ND	ND	3.720 ± 0.510	3.870 ± 0.608
	% Reduction			−8	−4
AFG_2_	No OG	ND	ND	0.090 ± 0.015	0.100 ± 0.040
	OG	ND	ND	0.070 ± 0.010	0.070 ± 0.016
	% Reduction			−22	−30

*Aflatoxin amount produced: μg per cm^2^ of fungal mat*.

a *ND, Not detectable*.

## Discussion

In this study, nine *Aspergillus* strains, namely, four toxigenic strains of *A. flavus* and *A. parasiticus*, two atoxigenic strains of *A. flavus*, and three *A. fumigatus* strains, WT and MAPK mutants (*sakA*Δ, *mpkC*Δ), were examined for their responses to different treatments. The *A. fumigatus sakA*Δ (*sakA* gene deletion) is an osmotic/oxidative stress sensitive mutant, while the *mpkC*Δ (*mpkC* gene deletion) is a mutant of the polyalcohol sugar utilization system (Xue et al., 2004; Reyes et al., 2006). In a prior study, both *sakA*Δ and *mpkC*Δ mutants showed higher sensitivity to certain benzo derivatives compared to the WT (Kim et al., 2011). The molecular biological/genetic resources (such as gene deletion mutants) for *A. flavus* or *A. parasiticus* available are few in number. Hence, *A. fumigatus sakA*Δ and *mpkC*Δ mutants could serve as model strains for investigating potential modes of antifungal responses in congeners, such as *A. flavus* or *A. parasiticus*. Except for the incapability to produce aflatoxins, the atoxigenic strains *A. flavus* 21882 and 18543 could produce KA (Table S1), a different type of secondary metabolite produced by *A. flavus* and *A. parasiticus*. Thus, the cellular secondary-product metabolism other than aflatoxin production is thought to be normal in the atoxigenic strains examined in this study.

Results showed that OG- or VT-based chemosensitization could enhance antifungal activity of Kre-Me in *Aspergillus* strains, where *Aspergillus* strains tested were sensitive to Kre-Me + OG or VT. OG was a more potent chemosensitizing agent than VT or BHAM to Kre-Me, where the concentration of OG necessary to achieve antifungal “synergism” was much lower (≥10 times lower) than the other compounds. When fungi are treated with Kre-Me, cellular AOX allows completion of electron transfer and ATP production via the MRC, thus resulting in overcoming the toxicity triggered by Kre-Me (or other MRC inhibitors) (Costa-de-Oliveira et al., 2012; Inoue et al., 2012). Therefore, AOX inhibitors, such as BHAM, have the effect to enhance the activity of Kre-Me (or other MRC inhibitors) when co-applied. The OG-based chemosensitization to Kre-Me, performed in this study, indicated that the AOX-inhibitory activity of OG (Sierra-Campos et al., 2009; Robles-Martinez et al., 2013) is much higher than that of the conventional AOX inhibitor, BHAM. Noteworthy is that the efficacy of OG-based chemosensitization to Kre-Me (which was “synergistic”) was higher compared to the prior chemosensitization test with “2-hydroxy-5-methoxybenzaldehyde (phenolic) + antimycin A (MRC inhibitor)” (Kim et al., 2011), in which the level of compound interaction was “additive/indifferent” but not “synergistic.”

The inhibition of MRC not only disrupts cellular ATP production, but also triggers oxidative stress, which results from abnormal leakage of electrons from the MRC (Fujita et al., 2004; Ruy et al., 2006). The escaped electrons cause oxidative damage to cellular components, such as cell membranes. Therefore, the enhanced oxidative stress generated by both MRC inhibitor (Kre-Me) and the redox-active phenolic derivative (OG) would result in increased growth inhibition of fungi.

Caffeine (a “control” reagent used in the cell wall/membrane integrity bioassay) tends to disorganize cell surface in fungi. Thus, fungi having abnormalities in cell surface integrity show increased sensitivity to caffeine treatment (Lussier et al., 1997). Caffeine also activates the protein kinase C (PKC) pathway, where the MAPK pathway genes *SLT2* and *BCK1* play key roles for maintaining cell wall integrity (Martin et al., 2000). Therefore, *SLT2* and *BCK1* gene deletion mutants (*slt2*Δ and *bck1*Δ) are hypersensitive to caffeine treatment, while caffeine sensitivity of *slt2*Δ and *bck1*Δ could be alleviated by sorbitol (Martin et al., 2000; Ferreira et al., 2006). As shown in this study, OG also disrupted cell wall integrity, where *S. cerevisiae* MAPK pathway mutants (*slt2*Δ, *bck1*Δ) showed enhanced sensitivity to OG, while this sensitivity was remedied by sorbitol.

OG possessed some moderate level of antiaflatoxigenic activity, where 4–30% of reduction in aflatoxin production was achieved depending on types of toxigenic *Aspergillus* strains. In a prior study, more than 95% of inhibition in aflatoxin production could be achieved with caffeic acid, which is another type of phenolic compound (Kim et al., 2008). Modulation of the expression of antioxidant genes, such as alkyl hydroperoxide reductases (Ahp1) that detoxify organic peroxides, has been the mechanism of antiaflatoxigenic activity of caffeic acid (Kim et al., 2008). However, caffeic acid did not exhibit potent antifungal activity in the same study, indicating antimycotoxigenic activity of phenolic compounds is not always parallel to their antifungal activity or *vice versa*. Although OG possessed potent antifungal activity, as determined in this study, its antiaflatoxigenic capacity is not comparable to other types of phenolic agents, such as caffeic acid (e.g., reduction of aflatoxin production: 4–30% w/ OG vs. >95% w/ caffeic acid). Therefore, the intervention of either aflatoxin production or the growth of fungal pathogens could be achieved by treating or modulating different cellular targets, such as antioxidant enzymes (Ahp1) for mycotoxin control or MRC and cell wall/membrane integrity, etc., for fungal growth control, respectively.

“Compound or chemosensitization—strain specificity” exists with OG, reflecting differential susceptibility of *Aspergillus* strains to the treatments. Of note, cellular signaling system, such as histidine kinase receptors, MAPK, etc., was involved in differential susceptibility of fungi to fungicide or drugs (Ochiai et al., 2002; Kojima et al., 2004; Chapeland-Leclerc et al., 2007). For example, (1) Three histidine kinase receptors were differentially involved in drug sensitivity or stress adaptation of the opportunistic yeast *Candida lusitaniae* (Chapeland-Leclerc et al., 2007), (2) The pathogenic yeast *C. albicans* (containing multiple histidine kinase genes) were sensitive to the fungicides iprodione and fludioxonil, while the model yeast *S. cerevisiae* (having only one histidine kinase gene) showed insensitivity to these fungicides (Ochiai et al., 2002), and (3) Intact MAPK signaling system is required for the effective antifungal activity of fludioxonil, where MAPK mutants of the fungal pathogen *Colletotrichum lagenarium* exhibited increased resistance to the fungicide. Thus, it is thought that (1) different set-up of signaling components in various species of *Aspergillus*, and/or (2) modification or mutation in cellular signaling system during evolution/stress adaptation could be possible mechanisms of actions for differential susceptibility of *Aspergillus* strains to fungicides or environmental stresses (such as Kre-Me tolerance of *A. fumigatus* MAPK mutants or heat responses, as determined in this study). Determination of precise mechanism of differential susceptibility to the treatments warrants future study.

As with agricultural application, the MRC is recently considered as a new antifungal target for clinical antimycotic therapy. Examples include: (1) MRC inhibitors increased fluconazole susceptibility of both patient and laboratory isolates of *C. albicans* (Sun et al., 2013); (2) co-application of antimycin A (another MRC-inhibitory drug) and BHAM significantly enhanced antifungal potency of posaconazole and itraconazole (Shirazi and Kontoyiannis, 2013), where the antifungal activity of the drugs was determined as “fungicidal” in mucormycosis-causing *Rhizopus oryzae*; (3) inhibition of MRC of *C. parapsilosis*, causative agent for neonatal and device-related infections, enhances susceptibility of the pathogen to caspofungin, which is a cell wall-disrupting drug [(Chamilos et al., 2006) and references therein].

Application of chemosensitizing agents, such as OG, would lower the effective dosage of MRC-inhibitory drugs or fungicides (e.g., strobilurins). This approach thus, lowers negative side effects of MRC inhibitors and/or prevents fungal tolerance to strobilurins, as described in this study. Consequently, lowered dosage level of antifungal agents would render treatment or agricultural practice, such as control of aflatoxin-producing *Aspergillus* strains, less expensive and safer.

In conclusion, OG and/or VT exhibit potential to serve as safe antifungal chemosensitizers that in concert with Kre-Me greatly potentiate antifungal activity. This capacity was shown to be effective in most of the *Aspergillus* strains tested in this study. Moreover, OG was shown to also have moderate antiaflatoxigenic activity.

### Conflict of interest statement

The authors declare that the research was conducted in the absence of any commercial or financial relationships that could be construed as a potential conflict of interest.
