# Supplementary material for: Use of benzo analogs to enhance antimycotic activity of kresoxim methyl for control of aflatoxigenic fungal pathogens
Source: Front Microbiol. 2014 Mar 7;5:87. doi: 10.3389/fmicb.2014.00087 (PMC3945611; doi:10.3389/fmicb.2014.00087)
Supplement: Supplementary file 1 [file DataSheet1.DOCX]

***Supplementary Material***

**Use of benzo analogs to enhance antimycotic activity of strobilurin for control of aflatoxigenic fungal pathogens**

**Jong H. Kim ^1*^, Noreen Mahoney, Kathleen L. Chan, Bruce C. Campbell, Ronald P. Haff and Larry H. Stanker**

^1^ Foodborne Toxin Detection and Prevention Research Unit, Western Regional Research Center, USDA-ARS, Albany, CA, USA.

*** Correspondence:** Jong H. Kim, Foodborne Toxin Detection and Prevention Research Unit, Western Regional Research Center, USDA-ARS, 800 Buchanan Street, Albany, CA, 94710, USA.

jongheon.kim@ars.usda.gov

1. **Supplementary Figures and Tables**

## Supplementary Tables

**Supplementary Table 1. KA assay in toxigenic and atoxigenic strains of *A. flavus*.** SD < 5%.

| *A. flavus* strains | 3357  Toxigenic | 4212  Toxigenic | 21882  Atoxigenic | 18543  Atoxigenic |
| --- | --- | --- | --- | --- |
| KA production  (mg mL^-1^) | 6.29 | 6.31 | 3.05 | 4.04 |

## Supplementary Figures


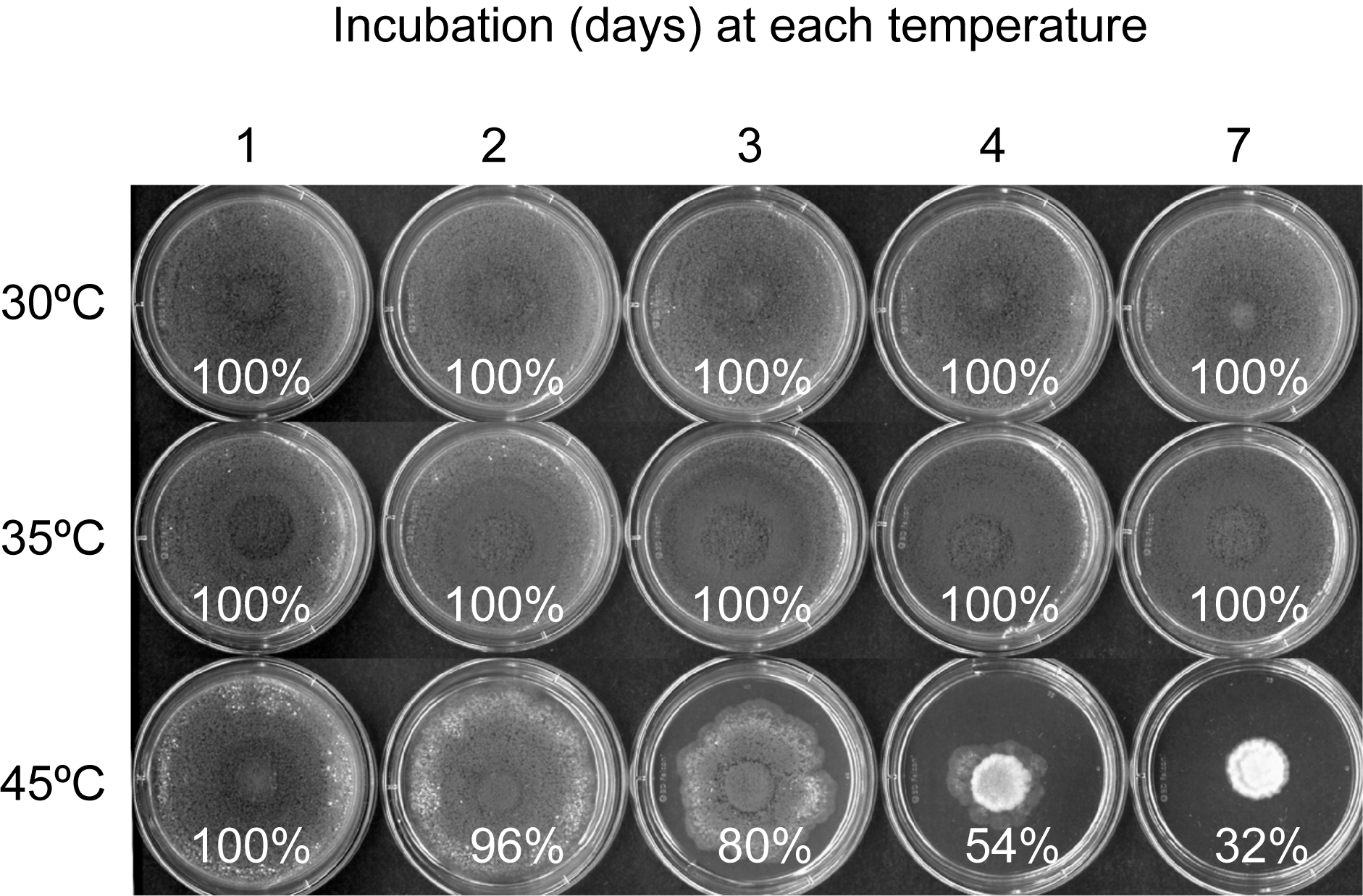


Supplementary Figure 1. Response of *A. flavus* 3357 to different temperatures. *A. flavus* showed sensitivity starting from 45°C, while it grew normally at 30 and 35°C. Similar result was observed with other *Aspergillus* strains tested except *A. fumigatus*, which was hyper-tolerant to heat treatment (up to 55°C; See Figure 2). 1 to 7, Incubation at each temperature for 1, 2, 3, 4 or 7 days, then growth recovery at 30°C for 6, 5, 4, 3 or 0 days, respectively.
